# The Task Matters: A Comprehensive Review and Proposed Literature Score of the Effects of Chemical and Physical Parameters on Embryo Developmental Competence

**DOI:** 10.3390/life13112161

**Published:** 2023-11-03

**Authors:** Alessandro Bartolacci, Francesca Tondo, Alessandra Alteri, Lisett Solano Narduche, Sofia de Girolamo, Giulia D’Alessandro, Elisa Rabellotti, Enrico Papaleo, Luca Pagliardini

**Affiliations:** 1 Obstetrics and Gynaecology Unit, IRCCS San Raffaele Scientific Institute, Via Olgettina, 60, 20132 Milan, Italy; alteri.alessandra@hsr.it (A.A.); degirolamo.sofia@hsr.it (S.d.G.); dalessandro.giulia@hsr.it (G.D.); rabellotti.elisa@hsr.it (E.R.); papaleo.enrico@hsr.it (E.P.); 2 Infertility Unit, Fondazione IRCCS Ca’ Granda Ospedale Maggiore Policlinico, 20122 Milan, Italy; francesca.tondo@policlinico.mi.it; 3 Reproductive Sciences Laboratory, Obstetrics and Gynaecology Unit, IRCCS San Raffaele Scientific Institute, Via Olgettina 60, 20132 Milan, Italy; solano.lisett@hsr.it (L.S.N.); pagliardini.luca@hsr.it (L.P.)

**Keywords:** temperature, oxygen, humidity, light, oil overlay, pH, embryo development, IVF outcomes

## Abstract

To explore the effects of chemical and physical parameters on embryo developmental competence, we conducted a systematic search on PubMed for peer-reviewed original papers using specific keywords and medical subject heading terms. Studies of interest were selected from an initial cohort of 4141 potentially relevant records retrieved. The most relevant publications were critically evaluated to identify the effect of these parameters on embryo development. Moreover, we generated a literature score (LS) using the following procedure: (i) the number of studies favoring a reference group was expressed as a fraction of all analyzed papers; (ii) the obtained fraction was multiplied by 10 and converted into a decimal number. We identified and discussed six parameters (oxygen, temperature, humidity, oil overlay, light, pH). Moreover, we generated a LS according to five different comparisons (37 °C vs. <37 °C; 5% vs. 20% oxygen; 5–2% vs. 5% oxygen; humidity conditions vs. dry conditions; light exposure vs. reduced/protected light exposure). Only two comparisons (37 °C vs. <37 °C and 5% vs. 20% oxygen) yielded a medium-high LS (8.3 and 7, respectively), suggesting a prevalence of studies in favor of the reference group (37 °C and 5% oxygen). In summary, this review and LS methodology offer semi-quantitative information on studies investigating the effects of chemical and physical parameters on embryo developmental competence.

## 1. Introduction

In the field of assisted reproductive technology (ART), human embryo culture plays a pivotal role in the success of in vitro fertilization (IVF) treatments. The delicate and intricate nature of preimplantation human development demands a meticulously controlled environment. During human embryo culture, chemical and physical parameters play a crucial role in embryo development and viability [1,2,3]. These parameters encompass a range of environmental conditions, including temperature, oxygen concentration, humidity conditions (HC), the use of oil overlay, and light exposure, all of which are carefully regulated within the laboratory setting. Moreover, these parameters directly influence the embryo metabolic activities [4,5,6,7,8,9,10,11,12,13,14,15,16,17,18]. It is well established that temperature ensures proper enzymatic reactions and cellular functions [4]. In addition, oxygen plays a vital role in supporting embryo metabolism and development [5]. While a consensus has been reached regarding the utilization of 5% oxygen levels compared to atmospheric levels (20%) [6], conflicting results have emerged when employing biphasic oxygen conditions (5–2%). Although the use of biphasic oxygen conditions appears to offer advantages in terms of blastulation, inconsistent findings have been reported in relation to clinical pregnancy [7,8].

Oil overlay has several important functions and benefits: (i) gas exchange, (ii) temperature stability, (iii) pH regulation, (iv) preventing contamination, and (v) minimizing disturbance. The inherent chemical and physical properties of the oil exert a significant influence on this vital aspect. These properties play a crucial role in shaping and determining the outcome, emphasizing the importance of understanding and considering them when working with human embryo culture [9]. Light exposure during mammalian embryo culture has garnered significant interest. However, despite several investigations, the impact of light on embryos remains a subject of ongoing debate, with inconclusive findings [10,11]. Recently, due to the introduction of dry incubators, several studies have investigated the impact of humidity conditions (HC) and dry conditions (DC) on IVF outcomes. While basic research studies show increased osmolality in culture medium under DC [12,13,14], these conditions do not seem to have negative effects on biological and clinical outcomes such as blastulation and pregnancy rates [16,17]. By carefully controlling these parameters, embryologists create an environment that mimics the natural conditions required for healthy embryo development. Nevertheless, despite these efforts, our culture conditions are unlikely to mirror precisely the dynamic environment experienced by embryos in vivo. Concerns exist that sub-optimal culture conditions could affect embryo developmental competence. Therefore, the meticulous quality control of these parameters is critical in maximizing the efficiency of our treatments. This comprehensive review explores the effects of chemical and physical parameters on mammalian embryo culture and their crucial roles in enhancing embryo development, implantation potential, and the overall success rates of IVF procedures.

## 2. Materials and Methods

### 2.1. Literature Search Methodology

A systematic search was conducted on PubMed to identify peer-reviewed original research articles related to the effects of chemical and physical parameters on development and clinical outcomes. The search strategy involved using relevant keywords and Medical Subject Heading (MeSH) terms. These keywords and Mesh terms were combined in various overlapping combinations to ensure the identification of publications specifically relevant to the topic: (“temperature” OR “oxygen” OR “humidity” OR “oil overlays” OR “light” OR “pH”) AND (“embryo quality” OR “IVF outcomes” OR “pregnancy” OR “live birth”). Furthermore, additional studies were identified by meticulously examining the reference lists of the selected publications. Full manuscripts were obtained for all the selected papers, and a thorough evaluation of the articles was conducted to make a final decision regarding their inclusion in the review. The most relevant publications, i.e., those concerning the effects of chemical and physical parameters on embryo development, as well as clinical outcome, were critically evaluated and discussed.

### 2.2. Study Selection

**Figure 1:**
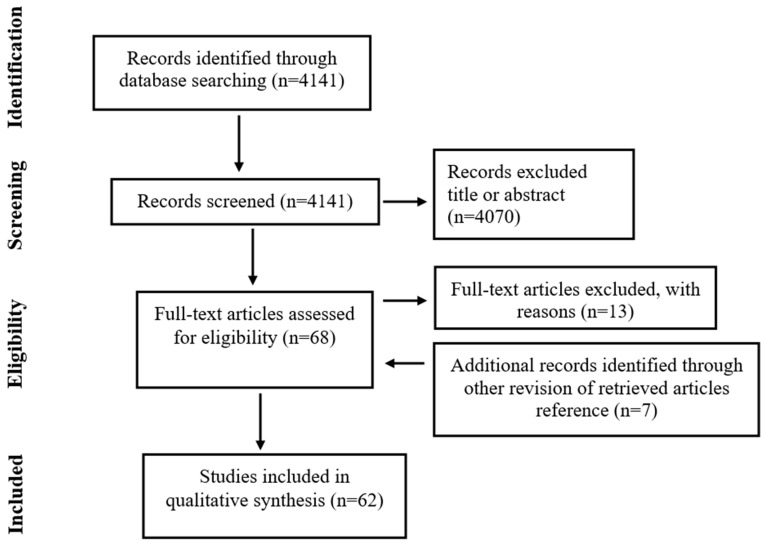
Flow diagram of study selection for review.

Two reviewers (AB and FT) independently assessed all studies for inclusion or exclusion. Disagreements were solved in discussion with a last author (LP). During the first screening, titles and abstracts were investigated and studies with a lack of any relevance were excluded; review articles were also excluded (Figure 1). The remaining articles were retrieved in their full length and assessed according to the eligibility criteria. The following information of such studies was collected: first author’s last name, year of publication, research objective, design of the study, outcomes investigated, and conclusions. No time restrictions were applied. Full-length articles were considered eligible if written in English. Data extraction was performed in 62 papers. A summary of the extraction results is shown in Table 1. In the following step, we generated a literature score (LS) using 4 parameters (oxygen, temperature, humidity and light) and 5 different comparisons [37 °C vs. <37 °C; 5% vs. 20% oxygen; biphasic oxygen (5–2%) vs. 5% oxygen; HC vs. DC; light exposure (LE) vs. reduced/protected LE]. The reference groups identified were HC for humidity, 37 °C for the temperature, LE for light, 5% and 5–2% for low vs. atmospheric (5% vs. 20%) and biphasic vs. monophasic (5–2% vs. 5%) oxygen, respectively. The LS was obtained from the percentage of papers reporting a positive correlation [improved outcomes for temperature, humidity, and oxygen (5% in 20% vs. 5% comparison and 5–2% in 5% vs. 5–2% comparison)] or negative correlation (compromised outcomes for LE) between a specific reference group and at least one biological/clinical outcome, as described below. Specifically, this score was calculated using the following procedure: (i) the number of studies favoring a specific reference group was expressed as a fraction of all analyzed papers; (ii) the obtained fraction was multiplied by 10 and converted into a decimal number. A score ranging from 1 to 5 indicates no evidence, 6 indicates low evidence, 7 indicates medium evidence, and 8–9 indicates high evidence of superiority for the reference group over the contrasting group. On the other hand, a score of 0 means that no study found a correlation between such reference groups and IVF outcomes, while 10 indicates that all studies converged towards a unanimous decision. To ensure consistency and reduce potential operator subjectivity in assessing outcomes, i.e., embryo quality, the authors collectively identified principal outcomes for analysis, focusing on objective measures such as fertilization, blastulation, euploid blastocyst formation, ongoing pregnancy, and live birth. Studies that did not report a correlation with the aforementioned outcomes were excluded from the LS calculation, but their findings were appropriately discussed if deemed relevant.

The major limitation of our LS is that it does not take into consideration the variable number of patients/embryos per study to which a different weight should be attributed. Moreover, another limitation is its inability to consider whether or not embryos are taken in and out of the incubator for embryo assessment. It is important to note that our approach does not seek to replace the results yielded using a meta-analysis, but rather serves as a complementary elaboration, enriching the written information presented in the manuscript.

## 3. Results

### 3.1. Oxygen

Oxygen plays a vital role in supporting embryo metabolism and development. In the female reproductive tract, oxygen concentration is typically around 2–8% [18]. Thus, in vivo, the oxygen concentration is different from the atmospheric levels. Several studies have investigated oxygen concentration during human embryo culture. One study showed higher blastulation, pregnancy, and live birth rates using 5% oxygen concentration [19], in contrast to another study that showed no improvements on fertilization, blastulation, and pregnancy rates [20]. Previous studies showed no significant difference in terms of fertilization, pregnancy, and implantation rates between 5% and 20% oxygen concentrations at the cleavage stage [21,22]. On the other hand, several studies showed higher top quality embryos, blastulation rate, and live birth in favor of 5% oxygen than 20% [23,24,25]. No difference was found in fertilization rate between 5% and 20% oxygen tension, but an increased number of top quality embryos on day 3, higher blastocyst formation, clinical pregnancy, and implantation rates in favor of 5% [26], according to one study that showed an overall increase in live birth when embryos were cultured in low oxygen tension [27]. Finally, a meta-analysis showed an improvement in the live birth rate of 43% during embryo culture in 5% oxygen concentration [6]. Accordingly, the latest recommendations provided from the ESHRE guidelines suggest the use of low oxygen concentration [1].

Interestingly, recent studies investigated the use of sequential oxygen tension (5% until day 3 and, subsequently, 2% from day 3 to day 5). This is probably to mimic the natural conditions of in vivo embryo development. A sibling zygote randomized control trial showed, although a small sample size, better blastulation rate when oxygen tension is reduced from 5% to 2% on day 3 for extended embryo culture (day 5) [7], in contrast to two studies that showed a similar blastocyst formation rate between 2%, 5% and 20% oxygen tension [8,28]. One report showed that blastocyst utilization rate is higher in 2% oxygen tension group [29], according to another study that showed improvement in blastocyst formation but only in low-quality human embryos cultured with 2% oxygen [30]. No significant difference were found between 5% and 3% oxygen tension in fertilization, blastulation and euploid blastocyst [31]. Recently, two studies suggested that biphasic oxygen culture could be an alternative strategy to increase the euploid blastocyst [32], blastocyst formation, and cumulative live birth rate [33].

We analyzed 18 studies for the LS calculation, 10 focused on comparing between 5% and a 20% oxygen concentration, resulting in a LS of 7. Additionally, eight studies examined the comparison between monophasic (5%) and biphasic (5–2%) culture oxygen tension, resulting in a LS of 5. These findings suggest there is no evidence that biphasic culture (5–2%) is better than monophasic culture (5%), especially in terms of clinical outcomes (Table 2).

### 3.2. Temperature

Maintaining the correct temperature is essential for proper gamete function and/or embryo metabolism and development [4]. Deviation from the optimal temperature can have detrimental effects on gamete function and embryo development, resulting in reduced viability and lower success rates in ART. Typically, the temperature is set at approximately 37 degrees Celsius (°C) to emulate the natural temperature found within the female reproductive tract. However, certain studies have suggested that a temperature of 36 °C may be more suitable to mimic the conditions of the female reproductive tract, potentially leading to improved fertilization and implantation rates [34,35]. Several studies have investigated the impact of temperature on IVF outcomes, yielding contradictory results. There has been evidence supporting negative consequences on the stability of the oocyte’s meiotic spindle when the temperature decreases [36,37], resulting in delayed embryo development [38], lower fertilization, and pregnancy rates [37]. A particular study found that the temperatures measured in the oviducts of non-mated, pre-ovulatory, peri-ovulatory, and post-ovulatory rabbits ranged from approximately 34.8 to 35.8 °C and from 35.9 to 36.6 °C in the sperm storage and fertilization site, respectively. These findings suggest that working at these temperatures (around 36 °C) may better mimic the human female reproductive tract [34], according to Higdon and colleagues, who showed a higher pregnancy rate when the incubator environment was cooler than 37 °C [35]. On the contrary, one randomized control trial showed that 36 °C does not improve embryo developmental competence and implantation rate [39]. A recent prospective sibling oocyte study suggests that culture temperature at 36.6 °C or 37.1 °C did not affect embryo development. However, it was observed that the clinical pregnancy rate was higher when the culture temperature was set at 37.1 °C [40], according to Fawzy and colleagues, who showed improvement in embryo development when the incubator was set at 37 °C [41]. Finally, one meta-analysis [42] showed no evidence that embryo culture at a lower temperature than 37 °C improves biological and clinical outcomes.

We analyzed six studies for the LS calculation, obtaining a high LS of 8.3 (Table 2), suggesting a prevalence of studies in favor of 37 °C.

### 3.3. Humidity Conditions

Humidity plays a significant role in the incubator environment. Maintaining optimal humidity levels is crucial to prevent excessive evaporation from the culture medium, which can affect embryo development by altering osmolality and pH [13]. However, it is important to acknowledge that humidity conditions in the incubator can have drawbacks as well. One notable concern is the increased risk of microbial contamination [12,71]. Advancements in IVF technology have led to significant improvements in incubator design. The latest generation of incubators now feature smaller individual chambers, specifically designed to minimize oscillations that may occur when the chambers are opened. However, the introduction of these new incubators, with their smaller individual chambers, has initiated a shift towards utilizing a DC atmosphere, as opposed to the conventional humidified environment. While this innovation offers advantages in minimizing oscillations during the opening of the chambers [72], there are concerns among scientists regarding the potential negative impact of DC on embryo developmental competence and clinical outcomes [15,43]. Two studies showed that significant evaporation occurs during single-step medium culture after 6 days in a dry incubator [14,44]. The humidity levels within incubators have a significant impact on the stabilization of osmolality [45], according to a previous study [46], suggesting that incubating the medium in a non-humidified environment leads to an increase in osmolality. The osmolality and pH of the culture media increase significantly over the course of 6 days of culture in both DC and HC, although the change was less with HC [13]. Nonetheless, evidence relevant to the impact of HC on biological and clinical outcomes are scarce and conflicting. A randomized controlled trial revealed a statistically significant decrease in implantation rates, as well as clinical and ongoing pregnancy rates, in DC [15], while another study found a difference in terms of ongoing pregnancy in the day 3 but not in the day 5 transfer policy [43]. Embryos developing under DC produced lower blastulation rates [47], in contrast to Valera and colleagues, who showed a comparable blastocyst formation rate and usable blastocyst [16]. The same authors showed a higher clinical pregnancy rate under HC in PGT cycles, but not in egg donation or autologous cycles. Moreover, the authors observed a negative impact of DC only on clinical pregnancy but not on ongoing pregnancy and live birth with use of single-step medium [16], according to a previous report that showed similar pregnancy and miscarriage rates [47]. Interestingly, another recent study using sequential medium yielded similar results. The authors suggest that HC do not enhance the rate of ongoing pregnancy and several embryological outcomes when employing a day 3 medium change-over [17]. These recent results [16,17] are reinforced by the control approach (the same incubator under two different conditions).

In conclusion, while basic research studies consistently indicate alterations in pH and osmolality of the culture medium under DC (although a relatively large volume of medium and a thick oil overlay cooperate in reducing evaporation), it is important to note that this consensus does not align with the clinical evidence. For the LS calculation, we analyzed four studies and obtained a LS of 5 (Table 2), suggesting no evidence of superiority for HC over DC.

### 3.4. Oil Overlay

In human embryo culture, an oil overlay is often used as a covering layer on top of the culture medium to create a specific environment for the embryos. One of the primary purposes of an oil overlay is to facilitate appropriate gas exchange within the embryo culture system, to minimize evaporation, and help maintain a stable environment. Despite this, evaporation could also occur with the use of a mineral oil overlay [44,46,73]. A study discovered that the osmolality of the medium (microdrops ranging from 50 to 200 μL) increased significantly when it was covered with mineral oil during a 5-day incubation period in a dry incubator. However, no such increase was observed when the incubation took place in a humidified atmosphere [14]. Furthermore, one study showed that one particular oil (oil B) exhibited a greater increase in osmolality compared to the three other oils (oils A, C, and D), which displayed similar increases in osmolality. This discrepancy can be attributed to the distinct physical oils composition. Specifically, oil B had lower viscosity and density, while its water content and activity were significantly higher [14]. Furthermore, denser oils have been observed to effectively reduce evaporation. In this context, a slight density difference of 0.04 g/mL can have a considerable influence on the rate of evaporation [46]. Another report indicated that using a 5 mL oil overlay resulted in lower osmolality compared to when only 3 mL was used [46]. Interestingly, a comparison of various brands of oil proposed that commercial oils exhibit variations in their ability to maintain the stability of osmolality and pH. Furthermore, the authors found differences in the total number of cells and the number of inner cell mass (ICM) of the obtained blastocysts across different oils [48]. To mitigate evaporation and prevent an increase in osmolality, employing a large volume of oil can effectively counteract these phenomena [45]. Indeed, higher evaporation occurs when using 3 mL of oil compared to using 5 or 7 mL in the same type of dish [73]. In this scenario, the volume of oil used to prepare the culture dishes plays a significant role in preventing medium evaporation and ensuring temperature stability. Using higher volumes of oil and ensuring a thicker layer can effectively minimize evaporation and maintain stable medium osmolality, particularly in single-step medium culture. Due to the specific inclusion criteria, calculating LS in relation to oil was not feasible.

### 3.5. Light

In vivo embryos, which develop inside the female reproductive system, are not directly exposed to light. However, during IVF treatments, embryos may be exposed to light, albeit in a controlled and regulated manner. There is scientific literature available on light exposure and its potential effects on embryo development; nevertheless, contradictory results have been obtained. In a study focusing on pre-implantation rabbit embryos, researchers found that subjecting the embryos to 24 h of visible light exposure did not lead to a significant increase in DNA ploidy abnormalities [49], in contrast to another study that showed how exposure to light for 24 h induced vacuolization, lamellar bodies, and increased electron density in the cytoplasm [50]. Moreover, the same authors suggested that the susceptibility of embryos to light might vary depending on their developmental stage [50]. Several studies have shed light on the potential effects of direct and prolonged exposure to visible light on oocyte’s rabbit. Light exposure does not interfere with the normal oocyte’s maturation process, embryos implantation, and cleavage rate [51,52,53]. A study examining pre-implantation rabbit embryos at different developmental stages investigated the effects of a 24 h exposure to light. The results of this study revealed contrasting outcomes for day 1 and day 3 embryos. In the case of day 1 embryos, exposure to light for 24 h led to noticeable cell degeneration, indicating a negative impact on their viability. On the other hand, day 3 embryos showed signs of apoptosis, albeit to a lesser extent compared to day 1 embryos. This suggests that the vulnerability to light-induced damage varies between different stages of embryo development [54]. Interestingly, one study, conducted on hamster and mouse embryos, showed that with just 3 min of exposure to microscope light, there was a significant increase in hydrogen peroxide levels [55]. Increased cytoplasmic electron density and fragmentation were found after an 8 h exposure to light [56]. Recently, white light has been reported to potentially decrease the implantation capacity of mouse embryos [11], in contrast to another two studies that showed no compromised fertilization rate, embryo development as well as clinical pregnancy with the use of a red filter light protection [57,64]. Moreover, prolonged exposure to light reduced the cleavage ability of rabbit embryos in a time-dependent manner, suggesting the use of red filtered light for prolonged exposure [58]. On the other hand, the use of a green filter on a microscope did not significantly improve bovine embryo development [59]. Two studies have investigated the probable harmful effects of blue light, showing that it has a negative impact on the blastulation rate of hamster and mouse embryos [60,61]. More recently, there has been evidence supporting the beneficial effects of yellow light irradiation on preimplantation development of mouse embryos during in vitro blastocyst production, regardless of the stage of the embryo [62]. Two studies investigated the potential detrimental effects of laser light on embryos, demonstrating no negative impact on embryo development, survival, and blastulation rates [10,63]. There exist differing perspectives regarding the potential adverse effects of light and our data suggest low scientific evidence for negative impacts with prolonged exposure to light. Moreover, we analyzed seven studies for the LS calculation, resulting in a low LS of 4.3 (Table 2).

### 3.6. pH

Culture media pH is a critical factor in human embryo culture. pH is closely correlated with carbon dioxide (CO_2_) levels due to the bicarbonate buffering system, in which changes in CO_2_ concentrations impact the production of carbonic acid, consequently leading to variations in pH. Adjusting the percentage of CO_2_ gas in the incubator is a fundamental method for precise pH control in the culture medium, which is essential for embryo development [65]. While embryos exhibit an impressive capacity to tolerate a wide range of pH values, it is crucial to note that deviations from the optimal pH range can have adverse effects on developmental competence [65]. In zygotes and embryos, intracellular pH (pHi) plays a pivotal role in maintaining cellular homeostasis, governing a myriad of cellular processes, including enzymatic reactions, cell division, and differentiation [65]. Fluctuations in the extracellular pH of the culture media directly influence the pHi of embryos, consequently affecting their homeostasis and developmental competence [69]. Although human embryos possess several intracellular mechanisms to regulate their pHi [66], any fluctuations can lead to cellular stress, impairing embryo developmental competence [67]. In comparison to embryos, oocytes exhibit heightened fragility due to their limited intrinsic capacity for robust pHi regulation, rendering them more susceptible to pH fluctuations [68]. Mammalian embryos at the morula and blastocyst stages appear to exhibit enhanced capabilities in regulating their pH levels due to the presence of tight junctions that are less permeable to H+ ions [69,70]. The optimal extracellular pH (pHe) was determined to be slightly higher than the pHi. Deviations in either direction, whether towards higher or lower pHe values, were observed to have inhibitory effects [66]. An ideal pH range of approximately 7.30 was identified for the pronuclear stage, followed by a lower pH value of 7.15 for cleaving embryos [71]. The pH of the culture medium pH can also be influenced by various additional factors, such as the laboratory’s geographical altitude. Altitude and air pressure can influence pH levels in embryo culture media due to variations in the solubility of CO_2_. Therefore, it is essential to consider altitude and air pressure to maintain a stable and optimal pH for embryo development.

## 4. Discussion

**Figure 2:**
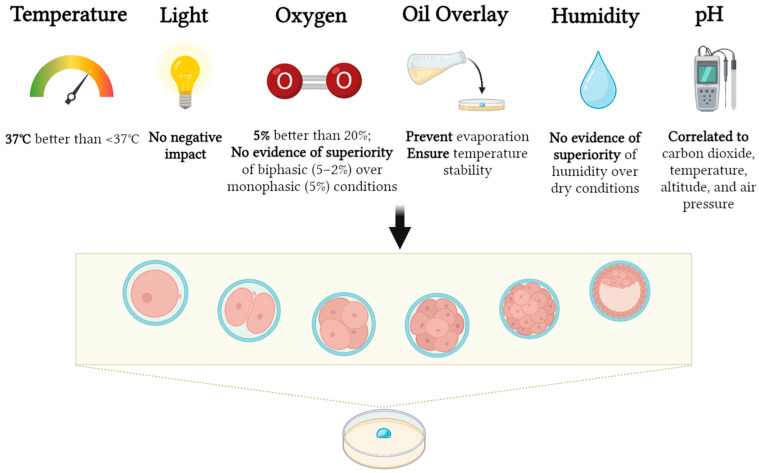
Summary of the main findings.

The purpose of this review was to evaluate studies focusing on the effects of chemical and physical parameters on mammalian embryo culture, with the aim of understanding their importance for human IVF treatments. Out of the 4141 initial studies, only 62 met the selection criteria. A summary of the main findings are shown in Figure 2.

Moreover, to provide further details, we have chosen to employ an alternative methodology. We generated a semi-quantitative outcome LS for the parameters that allowed it. Specifically, we identified four parameters (oxygen, temperature, humidity, and LE) and five different comparisons (37 °C vs. <37 °C; 5% vs. 20% oxygen; 5–2% vs. 5% oxygen; HC vs. DC; LE vs. reduced/protected LE) (Table 2). By adopting this approach, our intention was to bolster the objectivity of our conclusions, going beyond the limitations of a standard review. In relation to oxygen, we conducted an analysis of 18 papers that encompassed studies comparing low oxygen tension with atmospheric oxygen, as well as investigations into the efficacy of biphasic oxygen utilization. Numerous studies have consistently highlighted the advantages of employing low oxygen concentrations in embryo culture, particularly during extended culture periods [19,23,24,25]. These findings are supported by a recent meta-analysis, which demonstrated a substantial 43% improvement in live birth rates when embryos were cultured under low oxygen concentrations [6]. Notably, the latest recommendations from the ESHRE guidelines also endorse the use of low oxygen levels in embryo culture [1]. Regarding the use of low oxygen tension, we analyzed 10 studies, generating a LS of 7, suggesting medium evidence of superiority for 5% over 20% oxygen tension. On the other hand, assessing the efficacy of biphasic (5–2%) compared to monophasic (5%) culture, despite the use of biphasic oxygen, seems to have benefits in terms of blastulation; we obtained a low LS of 5, suggesting no evidence of superiority for biphasic over monophasic culture [29,30,31,32,33]. In our temperature analysis, we reviewed a total of 9 [34,35,36,37,38,39,40,41,42] studies, 6 of which were used to calculate the LS. We obtained a LS of 8.3, showing high evidence of superiority for 37 °C over cooler temperatures (36 < 37). According to a recent meta-analysis [42], our findings suggest that, currently, there is no compelling evidence supporting that embryo culture at temperatures lower than 37 °C leads to improved IVF outcomes. We analyzed 10 studies on humidity. It is evident that a humid environment plays a crucial role in reducing medium evaporation, leading to increased osmolality and pH [12,13,14]. While basic research studies show increased osmolality in culture medium under DC [12,13,14], these conditions do not seem to have negative effects on biological and clinical outcomes such as blastulation and pregnancy rates [16,17]. In the LS calculation, we analyzed four studies obtaining a low LS of 5, suggesting contradictory results (Table 2). Nevertheless, we analyzed a limited number of studies (four); consequently, further randomized controlled trials are needed to investigate this parameter. Due to the specific inclusion criteria, calculating the LS in relation to oil and pH were not feasible, but their findings were appropriately discussed. Our analysis of the papers related to oil emphasized its significance in preventing evaporation from the culture medium and in providing greater temperature stability. This crucial aspect is heavily influenced by the inherent properties of the oil, including water content, viscosity, and density. However, it is important to acknowledge that other factors, such as the culture conditions (humid or dry) and the volume of the drop medium, also play an important role in mitigating evaporation. Culture media pH is a critical factor in human embryo culture. While embryos exhibit an impressive capacity to tolerate a wide range of pH values, it is crucial to note that deviations from the optimal pH range can have adverse effects on developmental competence [65]. Furthermore, the pH of the culture medium can also be influenced by various additional factors, such as temperature [74] and the geographical altitude of the laboratory. For this reason, it is essential to consider temperature, altitude, and air pressure to maintain stable and optimal pH for embryo development. In our investigation into light exposure, we have examined 18 studies reaching contradictory results. Moreover, we analyzed seven studies for the LS calculation, resulting in a low LS of 4.3 (Table 2), suggesting limited clarity and no evidence regarding the potential negative impact of light on embryos, particularly in the context of human embryos. All studies on the toxic effects of light were experimental, so they may not accurately reflect real working conditions in an IVF lab. Furthermore, it is worth noting that a majority of these studies are conducted on animal models—i.e., rabbits—which may not be a good model reflecting human oocytes/embryos. Nevertheless, employing light filters can mitigate the adverse impact of light within IVF laboratories [57,58,59,60,61,62,63,64]. Moreover, it is important to note that the static nature of our current culture conditions does not accurately reflect the dynamic environment experienced by embryos in the human body [75,76]. Finally, in the longer term, large studies based on national birth registries are needed to clarify possible adverse effects for the newborn.

## 5. Conclusions

In summary, this review and proposed LS methodology offer semi-quantitative information on studies investigating the effects of chemical and physical parameters on mammalian embryo culture in order to minimize them in the practice of human IVF. Overall, we identified and critically discussed six parameters (oxygen, temperature, humidity, oil overlay, light, and pH). Furthermore, we generated a LS of five different comparisons (37 °C vs. <37 °C; 5% vs. 20% oxygen; 5–2% vs. 5% oxygen; HC vs. DC; LE vs. reduced/protected LE). Among these, two comparisons (37 °C vs. <37 °C and 5% vs. 20% oxygen) yielded medium-high literature scores, suggesting a prevalence of studies in favor of the reference group (37 °C and 5% oxygen). Conversely, the other three comparisons (5–2% vs. 5% oxygen, HC vs. DC, and LE vs. reduced/protected LE) produced a low score for 5–2% oxygen, HC, and LE.

## Figures and Tables

**Table 1:** Summary of the results from 62 papers identified in a review of the literature.

Study	Type of Study	Parameter	Comparison	Outcome	Results and Conclusions
Waldenström et al., 2009 [19]	Randomized	Oxygen	5% vs. 19%	LB	Improved in favor of 5%
Kea et al., 2007 [20]	Randomized	Oxygen	5% vs. 20%	OP	No significant difference
Dumoulin et al., 1995 [21]	Randomized	Oxygen	5% vs. 20%	OP	No significant difference
Dumoulin et al., 1999 [22]	Randomized	Oxygen	5% vs. 20%	OP	No significant difference
Ciray et al., 2009 [23]	Randomized	Oxygen	5% vs. 20%	BL	Improved in favor of 5%
Kovacic et al., 2008 [24]	Prospective	Oxygen	5% vs. 20%	BL	Improved in favor of 5%
Kovacic et al., 2010 [25]	Randomized	Oxygen	5% vs. 20%	LB	Improved in favor of 5%
Guo et al., 2014 [26]	Randomized	Oxygen	5% vs. 20%	LB	Improved in favor of 5%
Meintjes et al., 2009 [27]	Randomized	Oxygen	5% vs. 20%	LB	Improved in favor of 5%
Bontekoe et al., 2012 [6]	Meta-analysis	Oxygen	5% vs. 20%	LB	Improved in favor of 5%
Kaser et al., 2018 [7]	Randomized	Oxygen	5% vs. 5–2%	BL	Improved in favor of 5–2%
Yang et al., 2016 [8]	Experimental	Oxygen	5% vs. 20% vs. 2%	BL	No significant difference
De Munck et al., 2019 [28]	Randomized	Oxygen	5% vs. 5–2%	BL	No significant difference
Ferrieres-Hoa et al., 2017 [29]	Retrospective	Oxygen	5% vs. 5–2%	BL	Improved in favor of 5–2%
Li et al., 2022 [30]	Retrospective	Oxygen	5% vs. 5–2%	BL	Improved in favor of 5–2% in low quality embryos
Papadopoulou et al., 2022 [31]	Retrospective	Oxygen	5% vs. 3%	ER	No significant difference
Chen et al., 2023 [32]	Retrospective	Oxygen	5% vs. 5–2%	ER	Improved in favor of 5–2%
Brouillet et al., 2021 [33]	Retrospective	Oxygen	5% vs. 5–2%	BL, CLB	Improved in favor of 5–2%
Bahat et al., 2005 [34]	Experimental	Temperature	n.a.	TI	Temperature at the storage and fertilization sites are time and ovulation dependent
Higdon et al., 2008 [35]	Retrospective	Temperature	<37 °C vs. >37 °C	CP	Higher pregnancy at <37 °C
Zenzes et al., 2001 [36]	Experimental	Temperature	n.a.	SM	Alteration meiotic spindle at 0 °C
Wang et al., 2002 [37]	Experimental	Temperature	37 °C vs. 34 °C vs. 33 °C	CP	Higher pregnancy at 37 °C
Wang et al., 2001 [38]	Experimental	Temperature	n.a.	SM	Alteration meiotic spindle at <37 °C
Hong et al., 2014 [39]	Randomized	Temperature	37 °C vs. 36 °C	IR	Improved in favor of 37 °C
De Munk et al., 2019 [40]	Prospective	Temperature	37.1 °C vs. 36.6	CP	Improved in favor of 37.1 °C
Fawzy et al., 2018 [41]	Randomized	Temperature	37 °C vs. 36.5 °C	BL	Improved in favor of 37 °C
Baak et al., 2019 [42]	Meta-analysis	Temperature	37 °C vs. <37 °C	LB	Improved in favor of 37 °C
Chi et al., 2020 [43]	Retrospective	Humidity	HC vs. DC	OP	Improved in favor of HC
Fawzy et al., 2017 [15]	Randomized	Humidity	HC vs. DC	OP	Improved in favor of HC
Swain et al., 2018 [44]	Prospective	Humidity	HC vs. DC	OSM	Increased in DC
Yumoto et al., 2019 [14]	Experimental	Humidity	HC vs. DC	OSM	Increased in DC
Mestres et al., 2021 [45]	Experimental	Humidity	HC vs. DC	OSM	Increased in DC
Swain et al., 2016 [46]	Experimental	Humidity	HC vs. DC	OSM	Increased in DC
Holmes et al., 2018 [13]	Prospective	Humidity	HC vs. DC	OSM	Increased in DC
Del Gallego et al., 2018 [47]	Randomized	Humidity	HC vs. DC	BL	Increased in HC
Valera et al., 2022 [16]	Retrospective	Humidity	HC vs. DC	LB	No significant difference
Bartolacci et al., 2023 [17]	Retrospective	Humidity	HC vs. DC	OP	No significant difference
Yumoto et al., 2019 [14]	Experimental	Oil	Oil comparison	OSM	Increase osmolality in oil with lower viscosity and density, and higher water content
Swain et al., 2016 [46]	Experimental	Oil	3 mL vs. 5 mL	OSM	Lower in 5 mL group
Mestres et al., 2022 [48]	Experimental	Oil	Oil comparison	OSM	Increase osmolality in oil with lower viscosity and density
Swain et al., 2018 [44]	Prospective	Oil	Oil comparison	OSM	Increase osmolality in light oil
Mestres et al., 2021 [45]	Experimental	Oil	High vs. low volume	OSM	Increased in low volume
Schumacher et al., 1998 [49]	Experimental	Light	Light exposure	DP	No negative impact
Fisher et al., 1988 [50]	Experimental	Light	Light exposure	CI	Vacuolization, lamellar bodies after 24 h
Barlow et al., 1992 [51]	Experimental	Light	Light exposure	IR	No negative impact
Bedford et al., 1989 [52]	Experimental	Light	Light exposure	CP	No negative impact
Kruger et al., 1985 [53]	Experimental	Light	Light exposure	CR	No negative impact
Hegele-Hartung et al., 1988 [54]	Experimental	Light	Light exposure	ULTR	Negative impact on day 1 embryos; no impact on day 3 embryos
Nakayama et al., 1994 [55]	Experimental	Light	Light exposure	ORP	Negative impact
Hegele-Hartung et al., 1991 [56]	Experimental	Light	Light exposure	ULTR	Increased cytoplasmic electron density and fragmentation after 8 h
Li et al., 2014 [57]	Experimental	Light	Effect of red light	BL	No negative impact
Bognar et al., 2019 [11]	Experimental	Light	Light exposure	IR	Decreased implantation
Daniel et al., 1964 [58]	Experimental	Light	Light exposure	CR	Negative time-dependent impact
Korhonen et al., 2009 [59]	Experimental	Light	Green filtered exposure	ED	No negative impact
Oh et al., 2007 [60]	Experimental	Light	Blue light exposure	BL	Negative impact
Sakharove et al., 2014 [61]	Experimental	Light	Blue light exposure	BL	Negative impact
Jeon et al., 2022 [62]	Experimental	Light	Yellow light exposure	BL	Improved BL
Soares et al., 2014 [10]	Experimental	Light	Laser light	ED	No negative impact with use of low-level laser irradiation
Dinkins et al., 2001 [63]	Experimental	Light	Laser light	BL	No negative impact
Bodis et al., 2020 [64]	Prospective	Light	Light exposure	FR, BL, CP	Negative impact
Squirrell et al., 2001 [65]	Experimental	pH	Alteration pHi	ED	reduce ED
Phillips et al., 2000 [66]	Experimental	pH	pHi	pHi range	Mature oocytes: 6.98 ± 0.02; Cleavage stage embryos: 7.12 ± 0.01
Lane et al., 1999 [67]	Experimental	pH	Alteration pHi	ED	Impaired ED
Hentemann et al., 2011 [68]	Comparative study	pH	pH range	ED	7.30 before the pronuclear stage and pH 7.15 at the cleavage stage
Dale et al., 1998 [69]	Experimental	pH	pHi	FR, ED	Insemination in the human is pH-sensitive
Edwards et al., 1998 [70]	Experimental	pH	pHi	AM	30 mM DMO in the presence of non-essential amino acids and 1 mM glutamine did not block at the 2-cell stage

LB, Live birth; CLB, Cumulative live birth; CP, Clinical pregnancy; CI, Cell Injury; OP, Ongoing pregnancy; IR, Implantation rate; CR, Cleavage rate; ED, Embryo development; BL, Blastulation; ER, Euploidy rate; SM, Spindle morphology; OSM, Osmolality; HC, Humidity conditions; DC, Dry conditions; ORP, Oxygen radical production; VIS, Viscosity; DENS, Density; TI, Temperature identification; DP, DNA ploidy; ULTR, Ultrastructure; pHi, intracellular pH; AM, Amino-acids; DMO, 5,5-dimethyl-2,4-oxazol-idinedione; mM, millimolar; n.a., not applicable.

**Table 2:** Literature score of different chemical and physical parameters.

Parameters	Comparison	Reference Group	Studies in Favor of Reference Group	Overall Studies	Literature Score
Temperature	37 °C vs. <37 °C	37 °C	5	6	8.3
Oxygen	5% vs. 20%	5%	7	10	7
Oxygen	5–2% vs. 5%	5–2%	4	8	5
Humidity	HC vs. DC	HC	2	4	5
Light	LE vs. r/p LE	LE	3	7	4.3

HC, Humidity conditions: DC, Dry conditions; LE, Light exposure; r/p, Reduced/protected.

## Data Availability

Not applicable.
